# The Panel Spatial Econometric Analysis for Development of Green Intensive Agriculture Based on Edge Computing and Internet of Things

**DOI:** 10.1155/2022/2811119

**Published:** 2022-09-25

**Authors:** Qiubo Li, Hongyu Shi

**Affiliations:** ^1^Business School, Fuyang Normal University, Fuyang 236000, China; ^2^Business School, Henan Normal University, Xinxiang 453000, China

## Abstract

To further promote the modernization of agriculture and the prosperity of green industry, the analyses are made on the intensive level of agriculture by using spatial econometric model under the Internet of Things (IoT), and the optimal defense strategy is adopted for edge network equipment to ensure the security of agricultural information. Initially, the present work introduces the related concepts of agricultural intensive development and analyzes the important role of IoT in the development process of agricultural modernization. Next, it briefly explains the spatial econometric analysis method, introduces two basic spatial analysis models-spatial lag model (SLM) and spatial error model (SEM), and explains their principles in detail. Then, it signifies the characteristics of IoT and edge computing (EC) and designs the optimal defense strategy of edge network equipment from the perspective of IoT. Finally, the simulation experiment is carried out based on the edge network defense strategy, and the spatial econometric analysis is carried out by taking the agricultural intensive development of counties in a Chinese province as an example. The experimental results show that with the increase of the number of edge network devices, the optimal strategy of edge network defense can be adopted while consuming certain computing resources. The agricultural technology input and intensive level in the jurisdiction have high spatial correlation, so it is necessary to establish a spatial econometric model for analysis. Additionally, the statistics of SLM is higher than that of SEM, which shows that SLM can better reflect the technology investment and spatial correlation than SEM does. Both industrial and agricultural division of labor and agricultural production link division of labor can promote the level of intensification, among which the promotion of industrial and agricultural division of labor is not very significant, while the promotion of agricultural production link division of labor is very significant.

## 1. Introduction

The essence of the IoT is to let various sensing devices and intelligent terminals converge into a unified network system through a certain communication mode and connect the original independent carriers together, to realize barrier-free communication between things and people [1]. Edge computing (EC) is a hot term in recent years, which refers to the computing of data at the terminal close to the data source, the completion of traditional cloud data processing tasks at the edge, and the processing of computing and storage resources at the edge [2]. Traditional data processing mainly uses cloud computing. Now, it can sink to the edge, which will greatly reduce the pressure on the cloud. Of course, it can also be processed coordinated with other tasks. With the development and maturity of the IoT, the data volume of the central server is increasing. To reduce the huge computing pressure caused by the computing mode of a single mobile edge server, Lv et al. proposed a collaborative computing method to ensure the security and privacy of computer data [3]. For data collection or equipment monitoring, the ultimate purpose of data collection is to analyze the data. If the computing power of the equipment close to the data source allows, it can be carried out at the edge, which will greatly reduce the pressure on the cloud and a conclusion can be drawn quickly and efficiently. At present, EC has become an important part of the current information infrastructure, and its future development can be expected. Chen et al. believed that although the development of modernization had brought better life experience and great convenience to people's life, there were also many network-security risks, including information leakage and malicious network attacks. The current network security development cannot keep up with the urgent adoption of global smart city technology. Therefore, the method based on IoT and EC is very important to protect network information security [4].

Agricultural intensive development is an important part of modern agricultural development [5], which is the basic path to rapidly improve the agricultural development and is of great significance in the development process of industrialization and urbanization [6]. Agricultural intensive development is the basis of agricultural modernization. In recent years, the pace of industrialization and urbanization has left many high-quality land and agricultural labor resources stranded in the mountainous areas. It is very necessary to develop modern agriculture in the mountainous areas [7]. Hence, intensive usage of modern agricultural technique investment should be promoted. Product factors in the mountainous areas should be made use of to promote the intensive development of their agriculture. It can both stimulate the inner requirements of the modern agriculture in the mountainous areas and coordinate the pace of reform in the mountainous areas with the space of industrialization and urbanization. It has high significance for the modernized development in the mountainous areas [8].

To sum up, from the perspective of agricultural intensive development, the information security strategy is upgraded by means of IoT EC, and then, a spatial econometric analysis model is established. Based on the combination of IoT technology and spatial econometric analysis, it realizes the scientific analysis of green agricultural development and improves the agricultural division system of intensive development. Taking the counties under the jurisdiction of the province as the actual case, the SLM and SEM are used to verify and analyze the collected data, respectively. There is very important guiding significance for the development of agricultural modernization and the improvement of intensive level.

## 2. Agricultural Intensive Development in the Context of IoT

### 2.1. Related Concepts of Agricultural Intensive Development

“Extensive” and “intensive” are a couple of relative concepts, which refers to investing in the production of materials and labor on the same land area and adopting the method of expanding the cultivated land area to improve agricultural products in the present work [9]. Intensive production information and labor force investment refer to the unit product amount of the invested product materials and accomplishment of agricultural business in the same land area. The concept involves of two corresponding agricultural management methods of “extensive” and “intensive,” which is usually called intensive management of labor-intensive or intensive labor [10]. Extensive development and intensive development are two basic methods of economic development. Both methods are used by the agricultural economics department. Promoting the transformation of agricultural development into a broad intersection is both the inherent requirement of modern agricultural development and the basic path of transforming the mode of agricultural development. Agricultural intensive development is based on the operation mode of modern agricultural technology, which continuously improves the development process efficiency of agricultural strengthening level by promoting agricultural intensive enterprise activities and improving agricultural intensive operation effect [11].

Agricultural modernization is basically the modernization of science and technology, and agricultural production informatization is a direct reflection of the development of modern agriculture [12]. In the market exchange of domestic development, advanced agricultural knowledge should first be used in agricultural production [13] and agricultural information system. It is necessary to seamlessly mesh the advanced agricultural knowledge with other industry information systems, to realize agricultural modernization, including resource optimization and improvement of production, transportation, and sales efficiency [14]. The development of agricultural economy should improve efficiency and quality, reduce work intensity, and realize the sustainable development of agricultural production. Agricultural development contributes to the normal and stable development of domestic economy and plays an important role in promoting national competitiveness [15].

### 2.2. Overview of Spatial Econometric Analysis Methods

When there are similar values in a spatial field and there is a tendency of aggregation between these values, the spatial field will show a strong positive autocorrelation. On the contrary, if the spatial field has two values whose attributes are mutually exclusive, the spatial field will show negative autocorrelation [16]. Spatial autocorrelation represents the relationship between the observed values of two adjacent regions. The earliest spatial correlation is extended from spatial econometrics. After inheriting the classical panel econometric analysis method, the law is obtained by analyzing the spatial position and observation data between adjacent regions [17].

The spatial econometric model includes spatial autoregressive model (SLM) and SEM. The spatial autoregressive model mainly explains the spatial dependence caused by the interaction between variables such as spatial diffusion and spatial spillover, and the SEM is mainly used to reflect the impact of random impact on regional spillover [18]. There are usually two methods to establish spatial weight matrix: spatial weight matrix based on adjacency concept and spatial weight matrix based on distance. Assuming that the research object has *n* geographical regions, the spatial weight matrix of these *n* geographical regions can be expressed as

(1) $$\begin{array}{c} W_{n\times n}=\left\{\begin{array}{ccc} w_{11} & \cdots & w_{1n}\\ \vdots & w_{ij} & \vdots \\ w_{n1} & \cdots & w_{nn} \end{array}\right\}. \end{array}$$

In Equation (1), *w*_*ij*_ represents the neighbor relationship between area*i* and area *j*. There are two ways to determine its standard. Equation (2) denotes the calculation of *w*_*ij*_ in a spatial weight matrix based on neighbor concept.

(2) $$\begin{array}{c} w_{ij1}=\left\{\begin{array}{c} 1,\\ 0. \end{array}\right. \end{array}$$

In Equation (2), when area *i* is adjacent to area *j*, the value of *w*_*ij*1_ is 1, while, in other cases, its value is 0. Equation (3) signifies the calculation of *w*_*ij*_ in a spatial weight matrix based on distance.

(3) $$\begin{array}{c} w_{ij2}=\left\{\begin{array}{c} 1,\\ 0. \end{array}\right. \end{array}$$

*w*_*ij*2_ is equal to 1 when the distance between area *i* and the area *j* is less than *d*, and it equals to 0 in other cases. Spatial econometric analysis can explain the law in parameter value changings and influencing factors of a common attribute in two adjacent regions, and it can analyze the relationship between the two spaces, which is also the biggest difference between spatial econometric analysis method and general econometric model [19]. The spatial econometric model studies the change of spatial attributes between different regions. Although spatial econometric models have many setting forms, SLM and SEM are the most common basic models [20]. Initially, a univariate linear cross-sectional data regression model without spatial effect is established as follows:

(4) $$\begin{array}{c} y_{i}=\beta_{0}+\beta_{1}x_{i}+\varepsilon_{i}. \end{array}$$

In Equation (4), *y*_*i*_ is the observed value of the explained variable in the region, *x*_*i*_ stands for the observed value of the explained variable, *β*_0_ and *β*_1_ represent pending parameters, and *ε*_*i*_ refers to the random error [21]. Additionally, it is also necessary to transform the weight matrix in the spatial panel data and make the inner product of the matrix in time and the spatial weight matrix. Through the inner product transformation, the spatial panel data can be added with the time attribute [22]. Equations (5)–(7) illustrate the expressions of the transformed SLM and SEM.

(5) $$\begin{array}{c} y_{it}=\rho \left(I_{T}⊗W_{N}\right)y_{it}+\beta x_{it}+\varepsilon_{it}, \end{array}$$

(6) $$\begin{array}{c} y_{it}=\beta x_{it}+\varepsilon_{it}, \end{array}$$

(7) $$\begin{array}{c} \varepsilon_{it}=\lambda \left(I_{T}⊗W_{N}\right)\varepsilon_{it}+\xi_{it}. \end{array}$$

In Equations (5)–(7), *λ* measures the impact of the random error of the explained variables in adjacent areas on the explained variables in the area. As the basic form of spatial econometric model, although the SLM and SEM reveal the spatial effects of the explained variables and random error terms in adjacent areas on the explained variables in the area, they cannot explain whether the explained variables in adjacent areas have spatial effects on the explained variables in the area [23]. To make up for this defect, it is necessary to further expand the data of spatial panel analysis and analyze the spatial lag term of explained variables and explanatory variables according to the spatial Dobbin model:

(8) $$\begin{array}{c} y_{it}=\rho \left(I_{T}⊗W_{N}\right)y_{it}+\beta x_{it}+\theta \left(I_{T}⊗W_{N}\right)x_{it}+\varepsilon_{it}. \end{array}$$

In Equation (8), the purpose of spatial correlation test is to judge the selection of econometric model. Generally speaking, if the test results of correlation show that there is no spatial correlation, the ordinary econometric model is used for analysis. If there is spatial correlation, it is necessary to establish a spatial econometric model. Based on the ordinary least square regression of the panel data model without spatial effect, the Lagrange number multiplication (LM) statistics [24] is constructed. For the spatial correlation test of SLM and SEM, the calculations are shown in

(9) $$\begin{array}{c} \text{LML}=\frac{{\left[e^{.}\left(I_{T}⊗W_{N}\right)y/{\overset{\wedge }{\sigma }}^{2}\right]}^{2}}{J}, \end{array}$$

(10) $$\begin{array}{c} \text{LME}=\frac{{\left[e^{.}\left(I_{T}⊗W_{N}\right)e/{\overset{\wedge }{\sigma }}^{2}\right]}^{2}}{T\times T_{W}}, \end{array}$$

(11) $$\begin{array}{c} J=\frac{1}{{\overset{\wedge }{\sigma }}^{2}}\left[\left(I_{T}⊗W_{N}\right)x{\overset{\wedge }{\beta }}^{,}\left(I_{NT}-x{\left(x^{,}x\right)}^{-1}\right)\left(I_{T}⊗W_{N}\right)x\hat{\beta }+TT_{W}{\overset{\wedge }{\sigma }}^{2}\right], \end{array}$$

(12) $$\begin{array}{c} T_{W}=\text{tr}\left(WW+WW^{,}\right). \end{array}$$

In Equations (9)–(12), *e* represents the residual obtained after the ordinary least square's estimation of the panel data model without spatial effect, ${\overset{\wedge }{\sigma }}^{2}$ expresses the variance of the random error term, and tr denotes the trace of the matrix [21]. Besides, Equations (13) and (14) demonstrate the robust tests of the two models.

(13) $$\begin{array}{c} \text{robustLML}=\frac{{\left[e^{.}\left(I_{T}⊗W_{N}\right)y/{\overset{\wedge }{\sigma }}^{2}-e^{.}\left(I_{T}⊗W_{N}\right)e/{\overset{\wedge }{\sigma }}^{2}\right]}^{2}}{J-{TT}_{W}}, \end{array}$$

(14) $$\begin{array}{c} \text{robustLME}=\frac{{\left[e^{.}\left(I_{T}⊗W_{N}\right)e/{\overset{\wedge }{\sigma }}^{2}-{TT}_{W}/J\times e^{.}\left(I_{T}⊗W_{N}\right)y/{\overset{\wedge }{\sigma }}^{2}\right]}^{2}}{{TT}_{W}\left(1-{TT}_{W}/J\right)}. \end{array}$$

### 2.3. Overview of IoT and EC

The IoT is an integrated application of information technologies in the Internet era and one of the irreplaceable key technologies in the process of modernization [25]. Through the establishment of the IoT platform, applications like interconnection of everything and intelligent operation can be realized, such as information storage, automatic positioning, intelligent management, and monitoring. The emerging industry team, which is based on the IoT, is also growing, such as smart home furnishing, automatic driving system, face recognition, and smart city construction. Simultaneously, the development of intelligent system extends many popular fields, such as cloud database, data mining, intelligent analysis, and intelligent decision making [26]. Though the IoT has spawned many popular industries and research fields, because the volume of data is too large, it also produces many security problems. The processing of a large amount of data makes the center equipment of the network overwhelmed. The long response time of the system and the consumption of computing resources are also tough problems. To help the central server reduce the pressure, improve the data processing efficiency, and ensure the security and timeliness of data transmission, EC has become one of the important improvement methods [27]. EC refers to the centralized processing and analysis of data on the edge side away from the central server. It is similar to the central server and includes the functions of computing, storage, classification, and so on. Moreover, because edge devices are generally far away from the central server, the data is stored in the local server and does not occupy the memory of the central server, which avoids the risk of data leakage caused by malware attack in the process of data transmission and guarantees the data security and user privacy [28].

Cloud computing service is the core technology to help cloud database process and analyze data. Its main function is to use network transmission to migrate data to cloud database for centralized processing. Although cloud computing services can process and integrate data in batches, with the huge volume of cloud resources, centralized processing is not easy to achieve. On the one hand, it will increase the burden on the central server. Too much memory will also affect the running speed, resulting in too slow response time. On the other hand, a large number of centralized data storage and transmission will also produce huge security risks, which are the main defects of cloud computing services [29].

To make up for the deficiency of centralized cloud computing, the concept of EC comes into being. It refers to a distributed open platform integrating the core capabilities of network, computing, storage, and application near the edge of the network or data source to provide edge intelligent services nearby. Due to the shortening of the transmission link, EC can quickly and efficiently respond to business needs on the data generation side, and the local processing of data can also improve the degree of user privacy protection. Additionally, EC reduces the dependence of services on the network and can also provide basic business services in the offline state [30].

Compared with traditional cloud computing, EC has obvious advantages, as follows:

1. Low delay and high real-time performance

Generally speaking, the edge device is far away from the central server and at the boundary of the whole data system. Been put in another way to say, it is close to the data source. It can process the data immediately when receiving the data and then transmit it to the central server. EC's advantage is to reduce the pressure of the central server, speed up the data transmission time, and improve the operation efficiency of the central server.

(2) Less power consumption

The data preprocessing of edge devices can share part of the functions of the central server, greatly reduce the operation pressure of the central server and cloud database, and reduce the power consumption of network broadband [31].

(3) Reducing the risk of centralized data storage and improving the system's tolerance with fault

Since the data no longer needs to be uploaded to the EC for centralized processing, using EC can release some storage space and improve the operation speed of the system. When dealing with complex problems, it can free up more space, avoid the system locking phenomenon, improve the fault tolerance rate, and solve the security problem of data storage. Besides, because the edge device is far from the central server and the data is stored locally, the risk of data leakage can be greatly reduced.

(4) Security and privacy protection

EC can calculate and store the data on the edge device without uploading to the central server, so it can ensure the security and privacy of the data. Furthermore, EC can encrypt and desensitize the data through the key, which can realize the security authentication of the user's identity and ensure the privacy of the user's authority [32].

In general, EC can solve most of the problems existing in the IoT. With the help of EC, the IoT can develop faster and safer. Therefore, based on the large-scale edge network equipment, it is necessary to analyze the existence of the optimal solution. In the beginning, the total cost function of the edge network individual in time *t* is designed as

(15) $$\begin{array}{c} J_{i}=\min E\left\{\overset{T}{\underset{t=1}{\sum }}\left({\left|u_{i}\left(t\right)-f\left(r\left(t\right)\right)\right|}^{2}+\alpha_{i}{\left|x_{i}\left(t\right)\right|}^{2}\right)\right\}. \end{array}$$

In Equation (15), *u*_*i*_(*t*) represents the control variable when the edge device is optimal, *x*_*i*_(*t*) expresses the state variable, and *f*(*r*) describes the time-varying mean field term, which can be transformed by LM.

(16) $$\begin{array}{c} L_{1}\left(u_{1},..u_{n},\lambda \right)=E\left\{\overset{N}{\underset{i=1}{\sum }}\left({\left|u_{i}\right|}^{2}+\alpha_{i}{\left|x\right|}^{2}+\lambda ∙\frac{u_{i}}{N}\right)\right\}. \end{array}$$

In Equation (16), *λ* stands for the Lagrange multiplier, which can be obtained after further resolution.

(17) $$\begin{array}{c} L_{1}^{*}=\min E\left\{\overset{N}{\underset{i=1}{\sum }}\left({\left|u_{j}^{*}\right|}^{2}+a_{i}{\left|x_{j}^{*}\right|}^{2}+\lambda ∙\frac{u_{i}}{N}\right)\right\}. \end{array}$$

Then, *u*_*i*_^∗^ and *L*_1_^∗^ are the optimal solution of the function.

According to the above analysis, the average field game algorithm steps of network security defense based on the IoT edge device environment are as follows:

1. set the parameters: *x*, *a*_*i*_, *b*_*i*_, and *c* (parameter *a*_*i*_ indicates the influence factor of resource consumption of edge devices, and *b*_*i*_ refers to the influence factor of defense strength of edge equipment)
2. set the number of iterations
3. set time *T*
4. calculate the status track of edge equipment *x*_*i*_^∗^
5. calculate the optimal conditions and optimal solutions
6. analyze the relationship among optimal strategy, resource consumption, and attack intensity
7. end “For”
8. send back the track of cost function

### 2.4. Experiment Design

There are many influencing factors to be considered in the variable selection of spatial model. To make econometric analysis on the level of agricultural intensification, consideration should be both put into the specific quantification of these influencing factors, and whether the data is available and whether the source is reliable. These problems make the selection of control variables extremely difficult. After consulting the relevant literature and combining with the topographic characteristics of the studied area, the present work selects the control variables after comprehensive consideration: farmers' per capita income level; representative production factors of cultivated land area; the proportion of agricultural output value in the total output value of agriculture, forestry, animal husbandry, and fishery; per capita power consumption; and per capita total consumption.

Table 1 demonstrates the various control variables used in the spatial econometric model that analyzes the impact of the evolution of agricultural division of labor on the development of agricultural intensification.

The relevant data to calculate the above control variables are from the provincial data part of the *Statistical Yearbook*. A common panel data model is established based on these control variables:

(18) $$\begin{array}{c} J_{it}=\alpha +\beta_{1}F\left(1\right)+\beta_{2}F\left(2\right)+\beta_{3}F\left(3\right)+\beta_{4}I_{it}+\beta_{5}S_{it}+\beta_{6}E_{it}+\varepsilon_{it}. \end{array}$$

In Equation (18), *J*_*it*_ represents the agricultural technology investment in region *i*, during period *t*. *F*(1), *F*(2), and *F*(3), respectively, describes the division of agricultural industry, the division of agricultural products, and the division of agricultural production links in the region *i*, during period *t*. *I*_*it*_ denotes the income level of farmers, *S*_*it*_ refers to the characteristics of the production structure of the primary industry, and *E*_*it*_ stands for the scarcity of agricultural production factors.

Based on this model, the relevant programs in the spatial metrology toolbox are used in MATLAB software, and the Lagrange multiplication lag model (LML), Lagrange multiplication error model (LME), and robust test models (robustLML and robustLME) are adopted to carry out the spatial correlation test on the development of agricultural green intensification in 36 counties and districts under the jurisdiction of the province.

## 3. Experiment and Result Analysis

### 3.1. Defense Strategy Analysis of Edge Equipment

**Figure 1:**
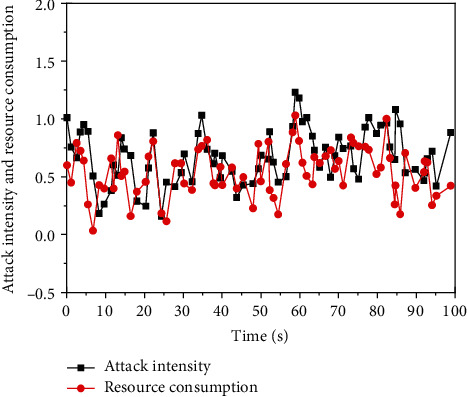
Relationship between the intensity of malicious attack on edge network devices and the consumption of computing resources.

Figure 1 manifests the relationship between defense response time and computing resource consumption of edge network devices.

Figure 1 indicates that when edge network devices are attacked by malware, the computing resource consumption of defense measures will change with the change of attack intensity. The higher the attack intensity, the higher the consumption of computing resources, but the overall maintenance is in a fixed interval. This is because edge network devices contribute some computing resources to deal with malware attacks to maintain the response time at a low level. With the increase of the number of edge network devices, the optimal strategy of edge network defense can be adopted while consuming certain computing resources.

### 3.2. Spatial Correlation Tests of Agricultural Intensive Development

**Figure 2:**
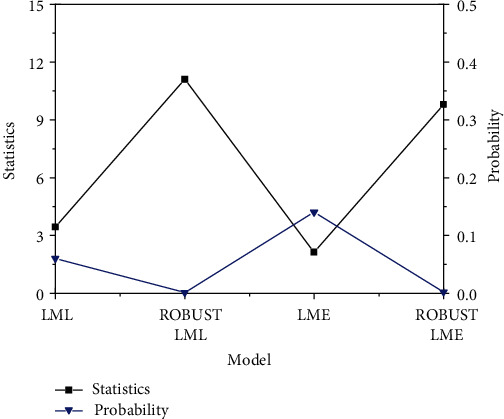
Results of spatial correlation tests of agricultural technology investment degree in investigated areas.

Figure 2 displays the spatial correlation test of agricultural development technology investment in the investigated province.

Figure 2 shows that the agricultural intensification level in the jurisdiction has high spatial correlation, so it is necessary to establish a spatial econometric model for analysis. Additionally, the statistics of LML are higher than LME. This shows that the SLM can better reflect the relationship between technology investment and spatial correlation than the SEM. Meanwhile, the robust LML statistic of stability test is higher than the LML of model test, indicating that the effect of stability test is more significant.

**Figure 3:**
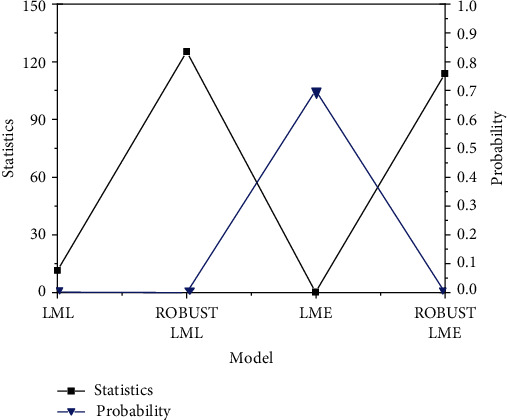
Results of spatial correlation test of agricultural intensification level in the areas.

Figure 3 demonstrates the spatial correlation test of agricultural development intensification level in this province.

Figure 3 shows that the agricultural intensification level in the jurisdiction has high spatial correlation, so it is necessary to establish a spatial econometric model for analysis. Additionally, the statistics of LML are higher than LME. This shows that the SLM can better reflect the relationship between technology investment and spatial correlation than the SEM. Meanwhile, the robust LML statistic of stability test is higher than the LML of model test, indicating that the effect of stability test is more significant.

Based on the SLM, the maximum likelihood estimation method is used to estimate the three fixed effect models, respectively. Figure 3 presents the final spatial results of econometric analysis.

**Figure 4:**
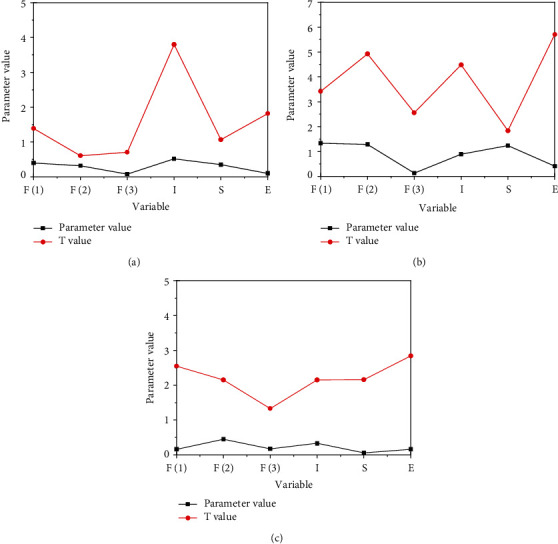
Results of spatial econometric analysis of agricultural production technology investment: (a) individual fixed effect, (b) time fixed effect, and (c) two-way fixed effect.

Figure 4 reveals that the estimation effect of the parameter values of the time fixed effect model is adverse, while the estimation results of the parameter values of the other two models are relatively significant. As for the fitting degree of the model, the fitting degree of the time fixed effect is the worst, and the fitting degree of the individual fixed effect is the best. Therefore, the individual fixed effect model is selected to analyze the intensification level. Figure 4 also bespeaks that both industrial and agricultural division of labor and agricultural production link division of labor can promote the level of intensification. Among them, the promotion from industrial and agricultural division of labor is not very significant, while the promotion from agricultural production link division of labor is very significant. The possible reason is that the division of labor between industry and agriculture is not obvious in the peasant class, and farmers can also engage in nonagricultural production process in the process of agricultural production. Under the condition of both, there is limited impact from industrial and agricultural division of labor on the level of agricultural intensification. For farmers, the balanced management of industry and agriculture is neither conducive to the production of agricultural products, nor able to meet the growing market demand. If the production model of self-sufficiency of agricultural products is maintained, the market transaction of agricultural products will stagnate. Even if the market demand is large and the technology investment is large, the intensive level cannot be improved. Moreover, farmers' balanced management cannot change the scarcity of means of production and factors of production. Although the production mode of “going out to work in idle time and going home to work in busy time” is a manifestation of reasonable time management and resource allocation for farmers and can also increase farmers' income to a certain extent, it cannot fundamentally change the scarcity of agricultural factors of production, which is impossible to realize the intensive development of agriculture and substantially to improve the quality of life of farmers.

For agricultural production, industrial and agricultural division, production link division, and agricultural product classification can promote agricultural technology investment. Among them, industrial and agricultural division and agricultural product classification have the highest significance, while the significance of production link division is lower. This may be because the division of labor between industry and agriculture promotes the transfer of productivity, and the transfer of labor can accelerate the internal digestion of the agriculture industry. The classification of agricultural products can accelerate the formation of a stable industrial chain and provide a realistic basis for technology investment. Because different types of agricultural products need different production technologies, specialized classification processing depends more on technology investment. Meanwhile, the income of most farmers is low, which makes farmers lack the ability to buy means of production, which is also one of the reasons for the overall low technology investment. The government's support and material subsidies can help farmers improve their technical investment to a great extent.

**Figure 5:**
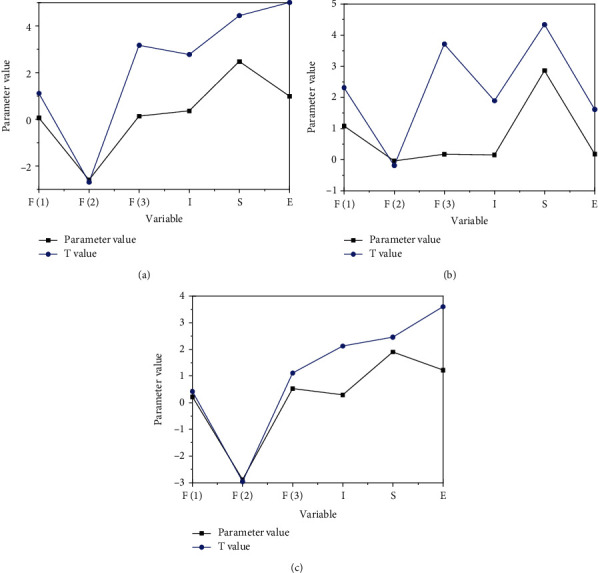
Spatial econometric analysis of agricultural intensification level in the jurisdiction: (a) individual fixed effect, (b) time fixed effect, and (c) two-way fixed effect.

Figure 5 illustrates the spatial econometric analysis results of agricultural intensification level.

Figure 5 reveals that the estimation effect of the parameter values of the time fixed effect model is adverse, while the estimation results of the parameter values of the other two models are relatively significant. As for the fitting degree of the model, the fitting degree of the time fixed effect is the worst, and the fitting degree of the individual fixed effect is the best. Therefore, the individual fixed effect model is selected to analyze the intensification level.

Figure 5 also bespeaks that both industrial and agricultural division of labor and agricultural production link division of labor can promote the level of intensification. Among them, the promotion from industrial and agricultural division of labor is not very significant, while the promotion from agricultural production link division of labor is very significant. The possible reason is that the division of labor between industry and agriculture is not obvious in the peasant class, and farmers can also engage in a nonagricultural production process in the process of agricultural production. Under the condition of both, there is limited impact from industrial and agricultural division of labor on the level of agricultural intensification. For farmers, the balanced management of industry and agriculture is neither conducive to the production of agricultural products, nor able to meet the growing market demand. If the production model of self-sufficiency of agricultural products is maintained, the market transaction of agricultural products will stagnate. Even if the market demand is large and the technology investment is large, the intensive level cannot be improved. Moreover, farmers' balanced management cannot change the scarcity of means of production and factors of production. Although the production mode of “going out to work in idle time and going home to work in busy time” is a manifestation of reasonable time management and resource allocation for farmers and can also increase farmers' income to a certain extent, it cannot fundamentally change the scarcity of agricultural factors of production, which is impossible to realize the intensive development of agriculture and substantially to improve the quality of life of farmers.

Additionally, there is a negative correlation between the type division of agricultural products and the level of intensification, which may be because the level of type division of agricultural products is not high, which hinders the development of agricultural intensification. Finally, there is a positive correlation between the agricultural intensive development level and space, indicating that the agricultural intensive level of the areas with higher intensive level is also higher in the adjacent areas. Hence, a regional collaborative development model was formed. The agricultural technology between different regions can spill over to the adjacent areas to promote the intensive development of regional agriculture.

## 4. Conclusion

Under the IoT, the present work uses the SLM and SEM to conduct panel spatial econometric analysis on the development of agricultural intensification and adopts the EC theory to defend the security of network equipment in the digital information environment, to ensure the information security in the development of agricultural modernization. Simultaneously, the security of defense strategy of edge network equipment is verified by simulation experiment, and the spatial econometric analysis of technology investment and intensive level development is carried out by taking the agricultural development in the province as an example. The results reveal that (1) when edge network devices are attacked by malware, the computing resource consumption of defense measures will change with the change of attack intensity. The higher the attack intensity, the higher the consumption of computing resources. (2) The level of agricultural technology input and intensification in the province has significant spatial correlation, which needs to be analyzed by establishing a spatial econometric model. (3) From the time fixed effect model, industrial and agricultural division of labor, production link division of labor, and agricultural product classification can promote agricultural technology investment. (4) From the individual fixed effect model, both industrial and agricultural division of labor and agricultural production link division of labor can promote the level of intensification, among which the promotion of industrial and agricultural division of labor is not very significant, while the promotion of agricultural production link division of labor is very significant. The deficiency is that the spatial econometric model proposed here only makes analyzation from the county level. To obtain accurate data, it also needs to be analyzed from the aspect of farmer. With the continuous development of IoT technology, EC will be more widely used, the data source of spatial measurement model will be timelier and more accurate, and the green intensive development of agriculture will be further improved.

## Figures and Tables

**Table 1:** Selection of control variables.

Research content of econometric model	Control variable selection	Control variable representation
The influence of the evolution of agricultural division on the degree of agricultural intensive management The influence of the evolution of agricultural division on the level of agricultural intensification	Farmers' income level Relative scarcity of agricultural production factors Characteristics of the production structure of the primary industry Agricultural infrastructure level Consumption level of urban and rural residents Characteristics of the production structure of the primary industry	Farmers per capita income level Representative production factors of cultivated land area The proportion of agricultural output value in the total output value of agriculture, forestry, animal husbandry, and fishery Per capita power consumption Per capita total consumption

## Data Availability

The raw data supporting the conclusions of this article will be made available by the authors, without undue reservation.
